# Level of Awareness About Dental Veneers and Their Oral Hygiene Care Among the General Saudi Public: A Cross-Sectional Study

**DOI:** 10.3390/healthcare13172170

**Published:** 2025-08-30

**Authors:** Diaa Almutairi, Saeed Alshahrani, Amwaj Balawi, Shahad Alnasser, Abeer Alshamlan, Hutham Almugim, Awatif Albalawi, Waheed Baig, Mohammad Alzahrani, Abeer Alaohali, Alanood Alqasim, Maha A. Alharbi

**Affiliations:** 1 Dental and Oral Health Department, Prince Sultan Military College of Health Sciences, Dhahran 34313, Saudi Arabia; amwajyounes@gmail.com (A.B.); shahadalnasser2@gmail.com (S.A.); huthamsalah@gmail.com (H.A.); awalbalawi@psmchs.edu.sa (A.A.); waheedbaig@psmchs.edu.sa (W.B.); jomaan@psmchs.edu.sa (M.A.); alohali@psmchs.edu.sa (A.A.); al.3anood12@hotmail.com (A.A.); harbimaha141@gmail.com (M.A.A.); 2 Department of Public Health, College of Applied Medical Sciences, King Khalid University, Abha 61411, Saudi Arabia; smsalshahrani@kku.edu.sa; 3 Prince Abdulrahman Advanced Dental Institute, Riyadh 11564, Saudi Arabia; dh.amsh@hotmail.com

**Keywords:** aesthetic dentistry, awareness, dental veneers, knowledge, oral health

## Abstract

**Background:** Dental veneers have gained growing interest recently as an aesthetic dental treatment. However, the public’s understanding of veneers’ benefits and risks is unclear. **Aim:** To assess the awareness and knowledge of the public about dental veneers in Saudi Arabia. **Methods:** This cross-sectional study employed an Arabic questionnaire, which consisted of three sections: demographic information, awareness about oral hygiene, and awareness about dental veneers. Correct responses to knowledge questions were assigned one point, and all points were summed to calculate the total knowledge score. **Results:** The study included 340 eligible respondents, out of whom 51 (15%) used veneers. Most responses to knowledge questions were correct. However, more than half of the participants wrongly thought that dental veneers are indicated for the correction of severely crowded teeth or to replace missing teeth, and 40.3% did not know that they require the removal of tooth structure. A low knowledge score about dental veneers was significantly related to male gender (*p* < 0.001), non-Saudi nationality (*p* = 0.005), attending medium/high schools only (0.014), and working in jobs outside the dental field (*p* = 0.036). **Conclusions:** The public’s knowledge regarding dental veneers in Saudi Arabia is fair but requires improvement to correct some misconceptions regarding the benefits and risks associated with the installation of veneers. The defects in knowledge identified by the present study should be addressed in patient education initiatives to improve patients’ understanding, align patients’ expectations, and help informed decision-making.

## 1. Introduction

Recent advances in dental techniques and technologies have contributed to the growing interest in aesthetic dentistry. One of the commonly employed dental aesthetic techniques is dental veneers [1]. Research has shown that the demand for teeth whitening rose significantly by 77.8%, while the public interest in dental veneers increased by 54.8% [2].

Veneer is a thin, customized layer made of ceramic or composite material. The tooth-coloured veneers are placed to cover the front surface of the teeth. Veneers can be used to address several dental issues, including worn enamel, misshapen teeth, minor misalignment of the anterior teeth, diastema closure, discolored teeth, and chipped or cracked teeth [3,4].

Veneers provided several benefits, including the improvement of the aesthetic appearance of the teeth, reduction in plaque accumulation, and the development of permanent stains, and better clinical longevity. In addition, veneers were widely and highly accepted by patients who regarded them as a safe dental treatment [5,6,7].

On the other hand, veneers have some disadvantages which may be encountered, including color mismatch with teeth, the potential for developing dentinal sensitivity, difficulties in repairing if fractured, and periodontal problems that arise with over-contoured veneers [5,8].

Numerous studies investigating public knowledge of oral health and dental prosthetics have reported less than optimal knowledge levels. A cross-sectional study that included 1332 participants from different Middle Eastern countries found that the general knowledge regarding dental veneers was unsatisfactory [9]. A meta-analysis that included 24 studies showed that patients with major systemic conditions worldwide have poor knowledge and awareness of the oral health associations with their condition [10]. Patients’ knowledge about veneers can contribute to patients’ expectations after the installation of dental veneers and is anticipated to influence the oral care exerted by the patient. Therefore, several studies have been conducted to assess the knowledge and attitudes of Saudi people towards dental veneers, reporting moderate or less than optimal knowledge levels [11,12]. Meanwhile, these studies did not investigate oral health knowledge and practices in relation to veneers.

The present study aimed to assess the level of awareness of dental veneers and the oral hygiene practices among the population of Saudi Arabia. In addition, the study aimed to identify the sources of information about dental veneers and the characteristics of individuals possessing poor knowledge about veneers to help define the targeted audience of health education strategies. This approach is grounded in the Knowledge-Attitude-Practice (KAP) theory, which emphasizes the role of awareness in shaping attitudes and behaviors, thereby guiding effective health education and promotion efforts [13]. We hypothesize that there are significant differences in the level of awareness of dental veneers across different demographic groups, and that there is a significant association between oral hygiene practices and awareness of dental veneers in the population.

## 2. Materials and Methods

### 2.1. Study Design, Setting, and Date

This cross-sectional survey study was conducted all over Saudi Arabia for two months. Ethical approval was obtained from Prince Sultan Military College of Health Sciences Institutional Review Board (Approval number IRB-2022-DOH-024). Consent was obtained from all participants after an explanation of the study’s objectives and methods. The confidentiality of the participants’ data was maintained by keeping the questionnaire anonymous and ensuring proper storage of the data, which was accessed only by the investigators and used for the current study purposes only.

### 2.2. Study Population and Target Group

Inclusion Criteria:

Participants eligible for this study were adults aged 18 years or older, proficient in Arabic, and currently residing within the geographical boundaries of Saudi Arabia at the time of data collection. Limiting the study population to residents ensures relevance to the local context, healthcare system, and cultural factors influencing awareness and practices regarding dental veneers.

Exclusion Criteria:

Participants were excluded if they provided incomplete or partially submitted questionnaires. Duplicated responses from the same individual were identified and removed to avoid bias. In addition, Saudi nationals residing outside Saudi Arabia during the study period were excluded, as they may have differing access to healthcare information and services, which could confound the study findings focused on the Saudi population. Additionally, responses from individuals failing to meet the language or age requirements were excluded.

### 2.3. Sample Size

The sample size was calculated using the following equation:

$$Sample\;size\;N=\frac{Z1-\frac{\alpha }{z2}p(1-p)}{d2}$$

where *d* = 0.05 (absolute error), *p* = 0.50 (expected response rate), and *Z* = 1.96 (Z-score for 95% confidence level). The minimum required sample size was 385 participants.

### 2.4. Sampling Technique

To identify the participants, the study used a non-probability snowball sampling technique across the five main regions of Saudi Arabia (Southern, Northern, Central, Eastern, and Western). The use of snowball sampling helps to reach out to as many representative samples as possible from the target population.

### 2.5. Outcome Assessment

Primary Outcome: To assess the level of awareness of dental veneers among the population in Saudi Arabia.

Secondary Outcome: To assess the knowledge and level of awareness about using ceramic laminate veneers in Saudi Arabia.

### 2.6. Data Collection

An online Google Form questionnaire composed of 40 questions was distributed electronically. Data collection took place for two months. The used questionnaire was derived from two validated questionnaires from previous studies [9,10], with modifications. We did not include the questions about the effect of the shade of resin cement on the final color and translucency of ceramic veneers, and the question about the removal of the dental plaque from the veneer being easier than removing it from the natural teeth [9]. We also did not include the questions about what preparation-less veneers and clip-on veneers are [10].

To ensure the scientific rigor of our study, the adapted questionnaire underwent a systematic process consistent with best practices in cross-cultural instrument development and validation. Firstly, the original questionnaire was carefully translated into Arabic using a forward-backward translation procedure by bilingual experts to ensure conceptual and semantic equivalence. Next, a pilot study was conducted with a representative sample of the target population (n = 20) to assess the psychometric properties of the Arabic version. Reliability was assessed by calculating Cronbach’s alpha coefficient, yielding a value of 0.631 (95% Confidence interval: 0.568–0.682). Furthermore, content validity was ensured through expert panel review involving subject matter specialists and Arabic linguistic experts to evaluate the relevance and cultural appropriateness of items.

The questionnaire was written in Arabic language and was composed of three sections. The first section inquired about the participants’ demographic data, such as gender, nationality, marital status, level of education, and which Saudi province they live. The second section was about the level of awareness of people regarding oral hygiene care for veneer placement (such as the frequency of tooth brushing and the use of floss or water floss). The third section assessed the general knowledge regarding dental veneers, such as the indications for veneers and their pros and cons.

One point was assigned for each correct answer in questions assessing knowledge, while incorrect answers received 0 points. The points for each participant were summed to calculate the total knowledge score, with a potential range between zero and 20 points.

### 2.7. Data Analysis

Complete responses were collected, and statistical analysis was carried out using the R Statistical language version 4.4.3 [14]. All the question responses were categorical, and they were presented as counts and percentages. The total knowledge score was summarized using the median, interquartile range (IQR, 25th–75th percentiles), and range, because normality testing based on the Shapiro–Wilk test, as well as kurtosis, indicated a deviation from normal distribution in some subgroups.

To compare the knowledge score across the categories of relevant participants’ characteristics, the Wilcoxon rank sum test (for comparing between two groups) and the Kruskal–Wallis test (for comparisons among more than two groups) were used. A multivariable robust linear regression model was carried out to assess the contributing factors to the knowledge score. A *p*-value of <0.05 was adopted for interpreting the results of statistical tests.

## 3. Results

A total of 500 individuals were invited to participate in this questionnaire, while 389 responses to the questionnaire were received. We excluded three respondents who declined to participate, and 47 who admitted to submitting a response to the same questionnaire. Finally, 340 responses were eligible to be included in this study. The response rate was estimated to be 68%.

Most respondents were young adults who belonged to the age groups “25–34 years” (20%) and “35–44 years” (32.6%). Most participants were female (80.9%), married (69.7%), had an academic degree (69.7%), and of Saudi nationality (96.8%). About one-third resided in the Western region of KSA (35.3%), while one-quarter resided in the Central region (25.9%). About 6% of the participants have worked in the dental field. Only 15% of respondents had veneers. The respondents heard about veneers from several sources, the commonest being dentists (48.5%), advertisements (41.5%), other people (31.8%), and the Internet (25.9%; Table 1).

About half of the 51 participants who had veneers used to brush their teeth twice daily before installing the porcelain dental veneers, while one-quarter brushed them once daily. Most participants with veneers used to wash their teeth after installing the dental veneers twice daily (45.1%) or more often (29.4%). Veneer-using participants tended to use mouthwash less frequently, with 41.2% using it less than once per day (Table 2).

About one-third of veneer-using participants did not use dental or water floss before or after installing the porcelain dental veneers. In addition, 29.4% stated that they did not receive instructions after installing dental veneers from the dentist or the dentist’s assistant (Table 3).

**Figure 1:**
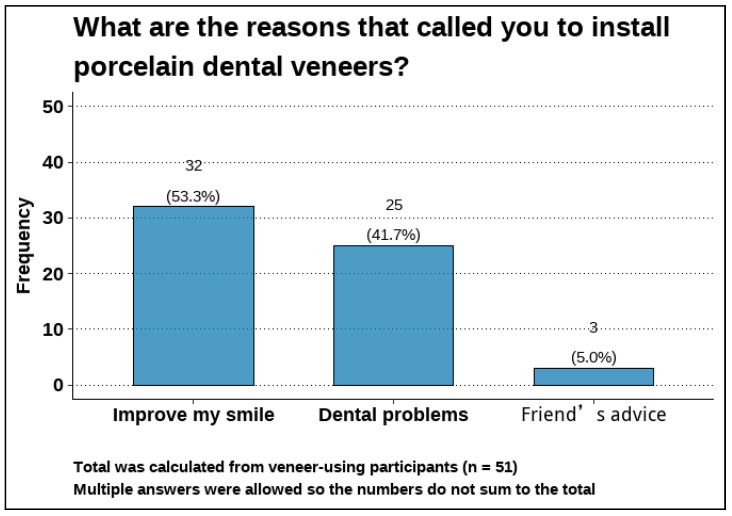
Reasons for installing dental veneers (n = 51).

The most reported reasons for installing dental veneers were improving the smile (53.3%) and the presence of dental problems (41.7%; Figure 1).

Most respondents thought that smoking or drinking coffee could affect the veneers (64.1%), while 27.6% thought that it could be avoided by following the right preservation. The majority of respondents knew that installing dental veneers does not keep them away from cleaning their teeth (92.1%; Table 3).

The knowledge of participants regarding the indications of dental veneers was mostly fair. However, more than half the participants wrongly thought that dental veneers are indicated for the correction of severely crowded teeth or to replace missing teeth. This misinformation was also represented in the participants’ answers to the benefits of dental veneers, where 71.2% thought that veneers are useful in correcting misaligned teeth, and 41.5% thought that veneers can prevent tooth decay/caries. As regards the disadvantages of veneers, a considerable proportion of participants (40.3%) did not know that it requires the removal of tooth structure. In addition, half the participants did not know whether regaining the original teeth was possible after the removal of veneers. One-third of respondents knew that several visits are required before the cementation, while 57.1% admitted their lack of knowledge (Table 4).

**Figure 2:**
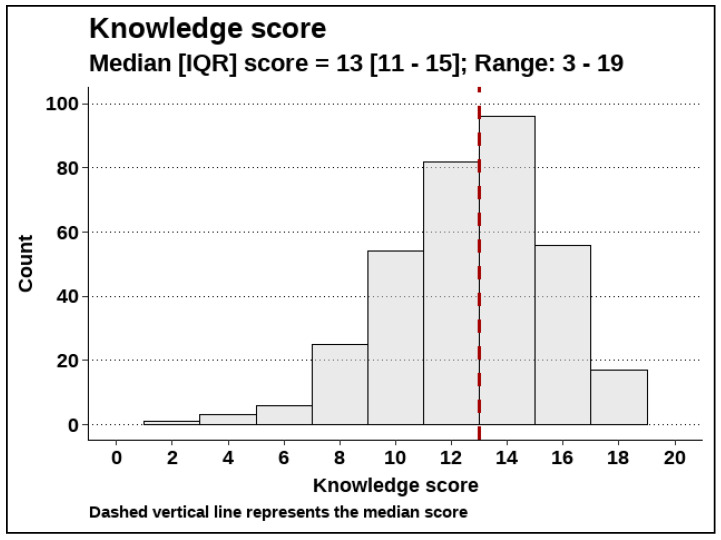
The total knowledge score (N = 340). Dashed Vertica; line represents the median score.

The total knowledge score was calculated by assigning one point to each correct answer in the questions presented in Table 4 and summing up the points for each participant. The median score was 13, with 50% of participants having a score between 11 and 15 (Figure 2).

Male participants had significantly lower values of the knowledge score compared to females (Median [IQR]: 11 [9,10,11,12,13,14] vs. 14 [12,13,14,15], *p* < 0.001). Non-Saudi participants also showed significantly lower scores compared to Saudis (Median [IQR]: 11 [10–11.5] vs. 14 [12,13,14,15], *p* = 0.005). Participants who attended only medium/high schools had significantly lower scores compared to those who attended university (Median [IQR]: 13 [10,11,12,13,14,15] vs. 14 [12,13,14,15], *p* = 0.014). Workers in jobs outside the dental field attained significantly lower scores compared to those working in the dental field (Median [IQR]: 13 [11,12,13,14,15] vs. 16 [12.5–18], *p* = 0.036). There were no significant differences in the knowledge scores in relation to age (*p* = 0.354), region of Saudi Arabia (*p* = 0.630), marital status (*p* = 0.520), or having veneers installed (*p* = 0.168). The effect size was small for all comparisons (Table 5).

A multivariable linear regression analysis was carried out to assess the contribution of the participants’ characteristics to the total knowledge score. The results showed that—after adjustment for all characteristics in the model—male gender was significantly associated with a decrease in the total knowledge score by approximately 2 points compared to females (adjusted B: −2.184, 95% CI: −3.079 to −1.289, *p* < 0.001). Also, Saudi individuals were significantly associated with a higher total score compared to non-Saudis (adjusted B: 1.988, 95% CI: 0.549 to 3.427, *p* = 0.007; Table 6).

## 4. Discussion

Dental veneers are among the most popular dental aesthetic techniques, which gained wide acceptance by patients due to their satisfactory aesthetic treatment and clinical longevity [1,6,7]. The present study aimed to assess the level of awareness of dental veneers and the oral hygiene practices among the population of Saudi Arabia. This study identified notable gaps in knowledge regarding dental veneers and revealed significant variations in awareness levels among different demographic groups. These findings underscore critical areas for targeted health education interventions. The subsequent paragraphs provide a detailed analysis of these gaps and their implications within the context of existing literature.

In the present study, most participants were female (80.9%). This finding was in line with previous questionnaire-based studies about dental veneers, which found that the majority of responses came from family participants [9,11]. This can be explained by the higher interest of women in their dental appearance than men [13]. However, one study from Al-Qassim region, Saudi Arabia, found that males constituted the majority (65%) of their sample [12].

Among our participants, the most commonly reported sources of information about veneers included dentists (48.5%), advertisements (41.5%), other people (31.8%), and the Internet (25.9%). This finding differs from other previous studies in Saudi Arabia, which reported different rankings of the participants’ sources of information. Asaad et al. [9] found that social media networks were the main source of information. Similarly, Alghamdi et al. [10] reported that the most common source was the internet and social media (56.3%), followed by friends and relatives (38.6%), then dentists (30.9%), and television (16.4%). Moreover, Alfouzan et al. [15], who included participants from different Middle Eastern nationalities, mainly Saudis, Kuwaitis, and Emiratis, found that the main source of information was the media.

As regards the reasons for installing veneers, the most reported reasons were improving smile (53.3%) and the presence of dental problems (41.7%). This result accorded with the findings of previous studies in Saudi Arabia [9,12] and in other Middle Eastern countries [15]. This reflects the dissatisfaction of a considerable proportion of people with their teeth’s appearance, which is supported by the results of previous research [16], which found that people tend to be dissatisfied with their tooth color and alignment. The high rate of dissatisfaction with dental appearance could be partially explained by the tendency of people to perceive their teeth’s shades as darker than they are [17]. Another potentially contributing factor is the influence of the media, as one study reported increased demand for veneers (54.8%) following the airing of makeover” television programs [2].

The knowledge level about veneers in the current study was mostly fair. The most important misconceptions that were identified included that “dental veneers are indicated for the correction of severely crowded teeth” (57.6%) or “to replace missing teeth” (65.3%), “veneers are useful in correcting misaligned teeth” (71.2%), “veneers can prevent tooth decay/caries) (41.5%). These misconceptions can lead patients to expect veneers to solve orthodontic or prosthetic issues that they cannot. This can result in disappointment, dissatisfaction with treatment outcomes, and potentially seeking inappropriate or ineffective treatments. Believing that veneers prevent decay or correct misalignment may lead patients to neglect necessary care, worsening issues like caries or malocclusion. This increases the risk of more complex and costly treatments later.

In addition, we found that 40.3% did not know that installing veneers requires the removal of tooth structure, and 50% did not know whether the original teeth can be regained after the removal of veneers or not. Not knowing that tooth structure must be removed for veneer placement may lead to underestimation of the invasiveness and permanence of the procedure. Patients may suffer regret or dissatisfaction if they are later faced with the fact that the natural tooth enamel has been irreversibly altered. Furthermore, 57.1% did not know how many visits were needed before the cementation, which might lead to incomplete or interrupted treatment, affecting the quality and durability of the veneers.

Previous studies also reported that knowledge about dental veneers was moderate at most in Saudi Arabia and identified various defects in knowledge. Asaad et al. [9] found that only 57% of their participants knew the need for tooth preparation before the cementation of veneers. Also, most participants did not know the potential effect of veneers on the gums (74%), whether veneers represented a permanent restoration or not (58%), how many visits are needed for the installation of veneers (77%), whether veneers can cause bad odor or not (74%), and whether removal of plaque was easier after veneer installation compared to natural teeth or not (60%).

Meanwhile, Alghamdi et al. [10] found that the total dental veneers knowledge was just above the score midpoint, indicating a moderate level of knowledge.

Alfouzan et al. [15] reported on the defects of knowledge among different Middle Eastern nationalities. The most notable incorrect information about dental veneers included those veneers are used for correcting severely crowded teeth (about 85%), anterior fractured teeth, do not need tooth brushing and dental flossing (about 97%), and prevent tooth caries and decay (about 93%). They also stated other important facts that only one third of the participants or less knew that veneers can be indicated for anterior fractured teeth (18%), and that they resist stains by beverages or smoking (33.5%), and that original teeth cannot be restored after removing veneers (9.8%).

On comparing the knowledge scores among the participants’ age groups, no significant difference was found (*p* = 0.354), which agrees with the results of Alghamdi et al. [10]. On the other hand, Alfouzan et al. [15] found that knowledge level varied according to the age groups, with the knowledge score decreasing with the advancement of age.

In the present study, Male participants had significantly lower values of the knowledge score compared to females (Median [IQR]: 11 [9,10,11,12,13,14] vs. 14 [12,13,14,15], *p* < 0.001). Likewise, Alghamdi et al. [10] stated that female participants had significantly higher knowledge scores than males (13.88 ± 3.16 vs. 13 ± 3.61, *p* = 0.003). Moreover, Alfouzan et al. [15] found that the mean score of correct responses was significantly higher in female participants (51.2 ± 18.7 vs. 44.4 ± 17.6, *p* < 0.001).

We found that non-Saudi participants had significantly lower scores compared to Saudis (Median [IQR]: 11 [10–11.5] vs. 14 [12,13,14,15], *p* = 0.005). In contrast, Alghamdi et al. [10] found a lack of significant difference in the total knowledge score between Saudi and non-Saudi participants. This discrepancy may be attributed to the higher educational level of Saudi participants in the current study compared to non-Saudis.

Educational level was related to the level of knowledge among our participants, as those who received only a medium/high school education had significantly lower scores compared to university graduates (Median [IQR]: 13 [10,11,12,13,14,15] vs. 14 [12,13,14,15], *p* = 0.014). This result is supported by the findings from previous similar studies. Alfouzan et al. [15] reported significantly higher mean scores of correct answers in participants with a university level of education compared to those with high school education or less (50.9 ± 18.5 vs. 45.3 ± 18.2, *p* < 0.001). Moreover, Alghamdi et al. [10] reported significantly higher mean scores in participants with university (14.03 ± 3.26) compared to participants with high school education (12.53 ± 3.28) or illiterate (10.63 ± 2.68) participants (*p* < 0.001). Similarly, in the study by Aljehani et al. [12], the participants who attained a higher educational level were more aware of the amount of care needed for veneers, the importance of avoiding rough manipulations, and the need for regular checkups and dental assessment. Individuals with higher educational levels are more prone to attain general knowledge and health-related knowledge and are more likely to pursue information from reliable sources.

We found no significant difference in the total knowledge score in relation to the marital status (*p* = 0.520). In partial agreement with our results, Alghamdi et al. [10] reported that a significant difference existed only between the scores of divorced participants (12.19 ± 3.69) and single participants (13.9 ± 3.21; *p* = 0.015), while no difference was found between single and married participants. In the present study, we did not inquire specifically about the widowed and divorced states, and they were presumably aggregated under the “single” status.

Our results indicated that working in the dental field was significantly related to the knowledge level, as workers outside the dental field had significantly lower scores compared to those working in the dental field (Median [IQR]: 13 [11,12,13,14,15] vs. 16 [12.5–18], *p* = 0.036), which is supported by the results of Alghamdi et al. [10] who reported higher scores for workers the dental field compared to those in other fields (15.86 ± 3.34 vs. 13.35 ± 3.26, *p* < 0.001).

Interestingly, we found that the level of knowledge did not significantly differ between the current users of dental veneers and those who never used veneers (*p* = 0.168). Likewise, Aljehani et al. [12] found that current users of veneers have comparable knowledge to non-users of veneers regarding disadvantages, lifespan, and proper care and cleaning of veneers. This lack of difference may reflect insufficient education of veneer users by their dentists or dentists’ assistants, which was elicited in the present study, where 29.4% of veneer users did not receive instructions before or after veneer installation. Dentists should provide all needed information to their patients regarding veneers’ indications, benefits, risks, and needed care to help inform decision-making by the patients, which will ultimately lead to higher satisfaction rates with the treatment and the treating dentists.

To prolong the life span of veneers, patients should adhere to good oral hygiene, including frequent tooth brushing, dental flossing, using mouthwash [18], as well as regular cementation of veneers [9]. As regards the oral hygiene measures among current veneer users, most participants continued to brush their teeth regularly after installing veneers. In addition, 41.2% did not use mouthwash was not used daily, and about 35% never used dental floss. This less-than-optimal adherence to oral hygiene measures could be attributed to the lack of knowledge among the participants, probably due to not receiving instructions from their dentists. This emphasizes the importance of dentists dedicating time to educate their patients about any dental interventions, even if the rate of complications is low.

This study provides valuable insights into the awareness, knowledge, and oral hygiene practices related to dental veneers within the Saudi Arabian population. The demographic profile of participants, predominantly female, aligns with previous research on dental aesthetics, suggesting a heightened interest in dental appearance among women [12,19,20,21]. While this trend is consistent, it contrasts with at least one regional Saudi study that observed a male majority [22,23,24,25], underscoring the potential for regional variations in study demographics and perhaps, differing patterns of engagement with dental aesthetic services.

Sources of information regarding dental veneers varied significantly from previous studies. This finding diverges from prior reports where social media and the internet featured more prominently as primary information conduits [26,27,28]. Similarly, Alfouzan et al. [15] identified media as the main source of information across a broader Middle Eastern cohort. These discrepancies highlight the evolving landscape of health information dissemination and consumption, emphasizing the need for targeted public health campaigns that leverage diverse communication channels.

The primary motivations for seeking veneers—improving smile and addressing existing dental problems are consistent with established literature [29,30,31]. This consistency reflects a pervasive dissatisfaction with natural dental aesthetics, a sentiment supported by research indicating common concerns about tooth color and alignment [32,33]. The influence of media, particularly “makeover” television programs, appears to significantly contribute to this dissatisfaction, driving demand for cosmetic dental procedures [34] such as veneers.

A crucial finding of this study is the generally fair level of knowledge about dental veneers among the participants, with several prevalent misconceptions. Significant knowledge gaps were identified concerning the indications for veneers (e.g., severe crowding or missing teeth), their role in correcting misaligned teeth, and their non-preventive effect on dental caries. Moreover, a substantial proportion of participants were unaware that veneer placement necessitates tooth structure removal or that original teeth cannot be fully regained post-removal. The lack of awareness regarding the number of required dental visits before cementation further highlights these knowledge deficits. These findings resonate strongly with previous studies that similarly reported moderate knowledge levels and specific areas of misunderstanding [35]. Cheung et al. [36] also noted widespread misinformation, particularly concerning the use of veneers for severely crowded teeth, their resistance to stains, and the need for ongoing oral hygiene.

No significant association was found between marital status and knowledge levels in this study, broadly consistent with previous work, although some distinctions between single and divorced participants have been noted elsewhere [37]. Unsurprisingly, individuals working in the dental field demonstrated significantly higher knowledge scores.

Finally, the assessment of oral hygiene practices among current veneer users indicated suboptimal adherence, with significant proportions not using mouthwash daily or dental floss consistently. This less-than-optimal compliance likely stems from the identified knowledge gaps and inadequate instruction from dental practitioners. This underscores the paramount importance of dedicated patient education by dentists regarding all dental interventions, even those with low complication rates, to ensure optimal long-term outcomes and veneer longevity [38,39].

The present study identified important defects in knowledge among the residents of Saudi Arabia regarding dental veneers and the required oral hygiene practices. The study results may be limited by the nature of survey studies, which depend on recall and thus may be prone to bias. Also, the sample technique did not follow probability sampling (randomization), which may lead to the inclusion of participants who possess the highest knowledge or interest regarding the topic of the study. Moreover, the cross-sectional nature of the study prevents the establishment of causality on investigating the factors associated with higher or lower knowledge scores. Also, the utilization of a web-based survey, while it improves reaching potential participants, can lead to under-representation of individuals with limited internet access or lower digital literacy, introducing selection bias and reducing generalizability.

## 5. Conclusions

In conclusion, this study found that the public’s knowledge regarding dental veneers in Saudi Arabia is fair but may require improvement to address some misconceptions about the benefits and risks associated with veneer installation. However, given the cross-sectional design and reliance on self-reported, web-based data, these findings should be interpreted with caution. The identified knowledge gaps highlight areas where patient education initiatives could be beneficial to improve understanding, align expectations, and support informed decision-making. Future interventions could consider tailored educational strategies, such as targeted awareness campaigns for male audiences, to help reduce gender-related disparities. Additionally, providing accessible, easy-to-understand materials aimed at individuals with lower educational levels may enhance comprehension and engagement. Further research is warranted to evaluate the effectiveness of such targeted campaigns, particularly concerning knowledge of oral hygiene maintenance after veneer placement, especially among potential candidates for the procedure.

## Figures and Tables

**Table 1:** Sociodemographic characteristics of the participants (N = 340). Multiple answers were allowed, so the numbers do not sum to the total.

Characteristic	N = 340
Age (Years), n (%)	
18–24	66 (19.4%)
25–34	68 (20.0%)
35–44	111 (32.6%)
45–54	82 (24.1%)
55 and more	13 (3.8%)
Gender, n (%)	
Female	275 (80.9%)
Male	65 (19.1%)
Nationality, n (%)	
Non-Saudi	11 (3.2%)
Saudi	329 (96.8%)
Which region do you live in? n (%)	
Western Region	120 (35.3%)
Central Region	88 (25.9%)
Eastern Region	58 (17.1%)
Southern Region	50 (14.7%)
Northern Region	24 (7.1%)
Marital status, n (%)	
Married	237 (69.7%)
Single	103 (30.3%)
Educational level, n (%)	
Medium and less	12 (3.5%)
High school	69 (20.3%)
Graduate	22 (6.5%)
Academic	237 (69.7%)
Do you work in the dental field, n (%)	
No	321 (94.4%)
Yes	19 (5.6%)
Did you have veneers, n (%)	
No	289 (85.0%)
Yes	51 (15.0%)
How did you hear about dental veneers, n (%) *a*	
Dentist’s recommendation	165 (48.5%)
Advertisements	141 (41.5%)
Talking to people	108 (31.8%)
Internet search	88 (25.9%)
Social media platforms	51 (15.0%)
Television	47 (13.8%)

**Table 2:** Participants’ practices regarding oral care. *a means* “*The total* was calculated from veneer-using participants (n = 51)”.

Question	More than Twice a Day	Twice Daily	Once a Day	Less than Once a Day
How many times do you brush your teeth before installing the porcelain dental veneers? *a*	9 (17.6%)	26 (51.0%)	13 (25.5%)	3 (5.9%)
How many times do you brush your teeth after installing the porcelain dental veneers? *a*	15 (29.4%)	23 (45.1%)	10 (19.6%)	3 (5.9%)
Do you use mouthwash? *a*	4 (7.8%)	9 (17.6%)	17 (33.3%)	21 (41.2%)

**Table 3:** Participants’ practices and opinions regarding dental veneers. *a means* “*The total* was calculated from veneer-using participants (n = 51)”.

Characteristic	N = 340
Do you use dental floss or water floss before installing the porcelain dental veneers, n (%) *a*	
Yes	17 (33.3%)
Sometimes	16 (31.4%)
No	17 (33.3%)
I’ve never heard of dental floss or water floss	1 (2.0%)
Do you use dental floss or water floss after installing the porcelain dental veneers, n (%) *a*	
Yes	17 (33.3%)
Sometimes	16 (31.4%)
No	17 (33.3%)
I’ve never heard of dental floss or water floss	1 (2.0%)
Were you given instructions after the installation of dental veneers by the dentist or assistant, n (%) *a*	
No	15 (29.4%)
Yes	36 (70.6%)
Do you think that smoking or drinking a lot of coffee affects the veneers, n (%)	
Affects, but with the right preservation, it can be avoided	94 (27.6%)
No	28 (8.2%)
Yes	218 (64.1%)
Do you think that when you install the dental veneers, it keeps you away from cleaning your teeth, n (%)	
No	313 (92.1%)
Yes	27 (7.9%)

**Table 4:** Knowledge about dental veneers (total N = 340).

Characteristic	N = 340
When do you think dental veneers are indicated, n (%)	
Badly stained teeth not responding to bleaching, n (%)	
No	87 (25.6%)
Yes *	253 (74.4%)
Correction of severely crowded teeth, n (%)	
No *	144 (42.4%)
Yes	196 (57.6%)
Replace missing teeth, n (%)	
No *	118 (34.7%)
Yes	222 (65.3%)
Anterior fractured teeth, n (%)	
No	118 (34.7%)
Yes *	222 (65.3%)
Multiple stained anterior restorations, n (%)	
No	87 (25.6%)
Yes *	253 (74.4%)
Dental fluorosis, n (%)	
No	104 (30.6%)
Yes *	236 (69.4%)
What are the considered benefits of dental veneers, n (%)	
Change tooth color, n (%)	
No	62 (18.2%)
Yes *	278 (81.8%)
Resist coffee/tea/smoking stains, n (%)	
No	120 (35.3%)
Yes *	220 (64.7%)
Do not require teeth brushing and flossing? n (%)	
No *	270 (79.4%)
Yes	70 (20.6%)
Change the tooth shape? n (%)	
No	64 (18.8%)
Yes *	276 (81.2%)
Prevent tooth decay/care? n (%)	
No *	199 (58.5%)
Yes	141 (41.5%)
Closure of the slight spaces between teeth? n (%)	
No	39 (11.5%)
Yes *	301 (88.5%)
Correction of maligned teeth that require orthodontic treatment? n (%)	
No *	98 (28.8%)
Yes	242 (71.2%)
What are the considered disadvantages of dental veneers? n (%)	
Require removal of the tooth structure, n (%)	
No	137 (40.3%)
Yes *	203 (59.7%)
May present an unpleasant odor (over contoured)? n (%)	
No	121 (35.6%)
Yes *	219 (64.4%)
May negatively affect the gums (over-contoured)? n (%)	
No	110 (32.4%)
Yes *	230 (67.6%)
May fracture due to a specific way of eating? n (%)	
No	68 (20.0%)
Yes *	272 (80.0%)
Require extensive care and hygiene? n (%)	
No	51 (15.0%)
Yes *	289 (85.0%)
If you decide to remove your veneers, can we have the original teeth as they were before? n (%)	
I don’t know.	170 (50.0%)
No *	136 (40.0%)
Yes	34 (10.0%)
Do you know how many visits are required before the cementation? n (%)	
I don’t know	194 (57.1%)
One visit	26 (7.6%)
Several visits *	120 (35.3%)

* Correct answer.

**Table 5:** Comparison of the knowledge score between subgroups of participants’ sociodemographic factors (N = 340).

Variables		Median Score	IQR of Score	*p*-Value c	Effect Size d
Age (Years)	18–24	13.50	11.00–15.75	0.354 a	0.001
	25–34	13.50	10.00–15.25		
	35–44	13.00	12.00–15.00		
	45–54	14.00	12.00–15.00		
	55 and more	13.00	10.00–13.00		
Gender	Female	14.00	12.00–15.00	<0.001 * b	0.281
	Male	11.00	9.00–14.00		
Nationality	Non-Saudi	11.00	10.00–11.50	0.005 * b	0.152
	Saudi	14.00	12.00–15.00		
Which region do you live in?	Central Region	13.00	11.00–16.00	0.630 a	−0.004
	Eastern Region	13.00	11.25–15.00		
	Northern Region	13.50	11.75–15.25		
	Southern Region	14.00	12.25–15.00		
	Western Region	13.00	11.00–15.00		
Marital status	Married	13.00	11.00–15.00	0.520 b	0.035
	Single	14.00	11.00–15.00		
Educational level	High school or less	13.00	10.00–15.00	0.014 * b	0.134
	University degree	14.00	12.00–15.00		
Did you have veneers?	No	13.00	11.00–15.00	0.168 b	0.075
	Yes	14.00	12.00–16.00		
Do you work in the dental field?	No	13.00	11.00–15.00	0.036 * b	0.114
	Yes	16.00	12.50–18.00		

IQR: Interquartile range (25th–75th percentiles). a Kruskal–Wallis test. b Wilcox rank sum test. c * Significant at *p* < 0.05. d Effect size (r for Wilcox rank-sum test and eta squared for Kruskal–Wallis test).

**Table 6:** Multivariable robust linear regression analysis for predictors of the total knowledge score.

Characteristic	Adjusted B (95% CI)	*p*-Value
Age (Years)		
18–24	—	
25–34	−0.607 (−1.650 to 0.435)	0.253
35–44	0.087 (−0.720 to 0.893)	0.833
45–54	0.038 (−0.821 to 0.897)	0.931
55 and more	−0.951 (−2.896 to 0.993)	0.337
Gender		
Female	—	
Male	−2.184 (−3.079 to −1.289)	<0.001 *
Nationality		
Non-Saudi	—	
Saudi	1.988 (0.549 to 3.427)	0.007 *
Educational level		
Medium and less	—	
High school	0.462 (−1.799 to 2.723)	0.688
Graduate	1.724 (−0.830 to 4.278)	0.185
Academic	1.049 (−1.102 to 3.200)	0.338
Do you work in the dental field?		
No	—	
Yes	1.068 (−0.864 to 3.000)	0.278
Did you have veneers?		
No	—	
Yes	0.279 (−0.684 to 1.242)	0.569

Abbreviation: CI = Confidence Interval. Adjusted R^2^ = 0.124; F = 4.05; *p*-value = < 0.001. * Significant at *p* < 0.05.

## Data Availability

The data presented in this study are available on request from the corresponding author due to ethical limitations that do not permit unrestricted public sharing of participant data.
